# Investigation of a *Staphylococcus argenteus* Strain Involved in a Chronic Prosthetic-Joint Infection

**DOI:** 10.3390/ijms21176245

**Published:** 2020-08-28

**Authors:** Alan Diot, Virginie Dyon-Tafani, Marine Bergot, Jason Tasse, Patricia Martins-Simões, Jérôme Josse, Florent Valour, Frédéric Laurent

**Affiliations:** 1 CIRI—Centre International de Recherche en Infectiologie, Inserm, U1111, Université Claude Bernard Lyon 1, CNRS, UMR5308, École Normale Supérieure de Lyon, Université Lyon, 69007 Lyon, France; alandiot@hotmail.fr (A.D.); virginietafani@hotmail.fr (V.D.-T.); marine.bergot@univ-lyon1.fr (M.B.); patricia.martins-simoes@chu-lyon.fr (P.M.-S.); florent.valour@chu-lyon.fr (F.V.); frederic.laurent@univ-lyon1.fr (F.L.); 2 BioFilm Control, 63360 Saint-Beauzire, France; jason.tasse@chu-lyon.fr; 3 National Reference Center for Staphylococci, Hospices Civils de Lyon, 69004 Lyon, France; 4 Regional Reference Centre for Complex Bone and Joint Infection (CRIOAc Lyon), Hospices Civils de Lyon, 69004 Lyon, France; 5 Department of Infectious Diseases, Hospices Civils de Lyon, 69004 Lyon, France; 6 Department of Bacteriology, Institute for Infectious Agents, Hospices Civils de Lyon, 69004 Lyon, France

**Keywords:** *Staphylococcus argenteus*, bone and joint infection, biofilm, intracellular, osteoblast

## Abstract

*Staphylococcus argenteus* is an emerging species responsible for infections comparable to those induced by *Staphylococcus aureus*. It has been involved in few chronic or persistent infections so far. In this study, we described a case of a persistent prosthetic-joint infection (PJI) affecting a young woman. We investigated in vitro the virulence traits of the incriminated *S. argenteus* strain (bone cell invasion, biofilm formation and induction of inflammation) and analyzed its genome, in comparison with two other strains of *S. argenteus* and two *S. aureus* isolates. It appeared that this *S. argenteus* PJI strain combined biofilm formation, osteoblast invasion and intracellular persistence abilities together with genes potentially involved in the escape of the host immune defenses, which might explain the chronicization of the infection.

## 1. Introduction

*Staphylococcus argenteus* is a novel and emerging species belonging to the *Staphylococcus aureus* complex and, as such, was historically misidentified as *S. aureus* clonal complex 75 [1]. It causes infections comparable to those triggered by *S. aureus*, e.g., skin and soft tissue infections, bacteremia and sepsis, and therefore can be life-threatening. Sporadic cases have been reported mainly in Asia, Oceania and Pacific islands [2,3] and more rarely in Europe [4,5].

*S. aureus* virulence relies on a large panel of toxins such as the Panton-Valentine leucocidin (PVL) or phenol soluble modulins (PSMs) as well as adhesion factors such as microbial surface components recognizing adhesive matrix molecules (MSCRAMMs) or various enzymes including proteases [6]. In addition, *S. aureus* infections can become chronic and last over months due to three major bacterial strategies: biofilm formation, non-professional phagocytic cell (NPPC) invasion [7,8,9,10] and switching to a quasi-dormant and slow-growing phenotype called “small colony variant” (SCV) [11]. These three virulence factors allow bacteria to escape both the immune system and the action of most antimicrobials. Internalization into NPPCs involves staphylococcal fibronectin binding proteins (FnBPs) that link fibronectin from the extracellular matrix acting as a bridge to the cellular β1 integrin [12,13,14]. Once into the cells, *S. aureus* can survive using the cell as a shelter to hide from extracellular antibiotics and the host immune system. In the same way, bacterial organization in biofilms offers a physical barrier against the immune system and antimicrobials [15]. In addition, it is now rather accepted that the reduced metabolism and growth rate associated with biofilm lifestyle, as well as changes in gene expression of biofilm-embedded bacteria, also provide a protection from both antibiotics and the immune system [16,17,18,19]. Factors and mechanisms responsible for *S. aureus* virulence have been extensively investigated but only few studies and data are available for *S. argenteus*. Genes encoding the PVL and staphylococcal enterotoxins have been identified [20,21,22], and a switch to a SCV phenotype during a chronic infection or in presence of high concentrations of amikacin has also been reported [23]. However, biofilm formation and cell invasion have not been extensively investigated yet in *S. argenteus*.

In the present study, we identified a strain of *S. argenteus* responsible for a chronic prosthesis-joint infection (PJI) in a young woman and assessed bacterial factors possibly responsible for the chronicization. After describing the clinical case, we investigated its in vitro phenotypical traits, i.e. cell invasion, biofilm production, cytotoxicity, induction of cytokines secretion as well as its genotypical traits. Results were compared with those obtained with two other strains of *S. argenteus*, as well as two *S. aureus* strains used as references.

## 2. Results

### 2.1. Clinical History

**Figure 1:**
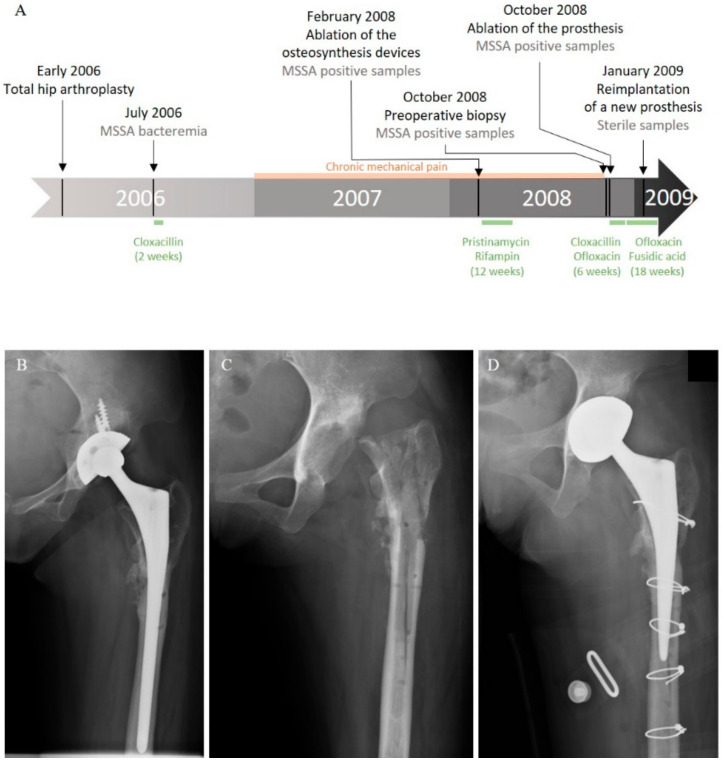
Clinical history of the case. (**A**) Schematic representation of the course of the infection and treatments. Left hip X-ray: (**B**) after osteosynthesis device ablation; (**C**) after prosthesis removal; and (**D**) after reimplantation.

In 2006, a 20-year-old Cambodian woman benefited from a total hip arthroplasty with shortening subtrochanteric osteotomy for a left hip osteoarthrosis secondary to a developmental dysplasia. A hip prosthesis and osteosynthesis devices (plates and screws) were implanted in the femur. Postoperative course was marked by a bacteremia originating from a peripheral catheter-related phlebitis due to a bacterium identified at this time as a methicillin-susceptible *Staphylococcus aureus* (MSSA). The patient was treated by a two-week course of intravenous cloxacillin (Figure 1A). Six months later, she developed chronic mechanical pain of the left hip. In February 2008, after more than a year of mechanical pain, the ablation of osteosynthesis devices was performed with prosthesis retention (Figure 1A,B).

Systematic periprosthetic bone tissue samples yielded few colonies of a bacterium identified as MSSA. A combination of pristinamycin and rifampin was prescribed for three months. As pain worsened, a two-stage exchange strategy was planned in October 2008 (Figure 1A,C). Bone tissue sampled before the surgery and during the prosthesis ablation were still positive for MSSA (Figure 1A). The postoperative treatment using intravenous cloxacillin and oral ofloxacin was continued for six weeks, and then switched to an oral combination of ofloxacin and fusidic acid. A prosthesis reimplantation was performed after three months of treatment (Figure 1A,D). Bacteriological samples remained sterile at that time. Antimicrobial therapy was stopped three months later, and, after a five-year follow-up, the patient was doing well, with no clinical or radiological symptoms of recurrence.

The strain isolated during the last episode of recurrence was originally identified as a methicillin-sensitive *Staphylococcus aureus* using Vitek2 (Biomérieux) for identification and antibiotic susceptibility determination. The sequence type of this strain was defined as ST75, using DNA microarrays and identibac *S. aureus* Genotyping (Alere) [24,25]. However, when its genome was sequenced for the need of a recent research project, we acknowledged this strain as belonging to the *Staphylococcus argenteus* species. Since all the strains identified during this case were MSSA harboring the same antimicrobial susceptibility profile (Table 1), we wonder if all the strains could actually be *S. argenteus* misidentified as MSSA. However, we cannot clarify this point as we cannot sequence the isolates from the previous episodes of infection because the samples are no longer available.

This case would thus be one of the few chronic *S. argenteus* infections described and we decided to investigate the cell invasion and the related cellular response, as well as the biofilm production properties of this strain (*S. argenteus*^PJI^) using in vitro tests.

### 2.2. Cellular Invasion Properties

**Figure 2:**
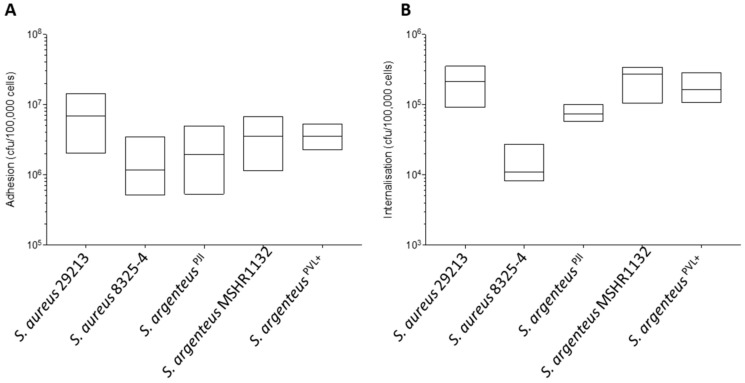
Properties of adhesion and internalization of the *S. argenteus*^PJI^ strain. (**A**) The adhesion to MG63 cells after 2 h of contact of the *S. aureus* and *S. argenteus* strains is expressed in CFU/100,000 cells. *S. argenteus*^PJI^ adhere to the cells with a similar efficacy than all the strains tested. (**B**) The intracellular inoculum recovered after 2 h of contact and 1 h of gentamycin treatment to kill the extracellular bacteria is expressed in CFU/100,000 cells. The *S. argenteus* ^PJI^ responsible for the chronic PJI showed an intermediate internalization between the poorly invasive *S. aureus* 8325-4 and the invasive *S. aureus* 29213 or *S. argenteus* MSHR1132, *S. argenteus*^PVL+^ strains.

We first assessed the capacity of *S. argenteus*^PJI^ to adhere and invade non-professional phagocytic cells, osteoblastic MG63 cells. Our results show an ability for cellular adhesion that appears similar to that of the poorly invasive *S. aureus* 8325-4 (Figure 2A, 2.44 ± 1.84 vs. 1.69 ± 1.23 × 10^6^ CFU) and slightly weaker than the one of the highly invasive *S. aureus* 29213 (Figure 2A, 2.44 ± 1.84 vs. 7.80 ± 4.82 × 10^6^ CFU).

When compared to the other *S. argenteus* strains, the cellular adhesion was also in the same range (Figure 2A, 2.44 ± 1.84 vs. 3.79 ± 2.21 and 3.65 ± 1.12 × 10^6^ CFU for *S. argenteus* MSHR1132 and *S. argenteus^PVL+^* respectively).

At 3 h pi, *S. argenteus*^PJI^ was internalized more efficiently into MG63 cells than *S. aureus* 8325-4 (Figure 2B, 7.47 ± 1.46 vs. 1.25 ± 0.59 × 10^4^ CFU for *S. argenteus*^PJI^ vs. *S. aureus* 8325-4). However, intracellular bacterial load appeared lower than those of *S. aureus* 29213 (Figure 2B, 7.47 ± 1.46 vs. 20.78 ± 9.04 × 10^4^ CFU) or of the other *S. argenteus* strains that also proved effective at invading cells (Figure 2B, 7.47 ± 1.46 vs. 23.37 ± 8.98 and 15.78 ± 5.35 × 10^4^ CFU).

**Figure 3:**
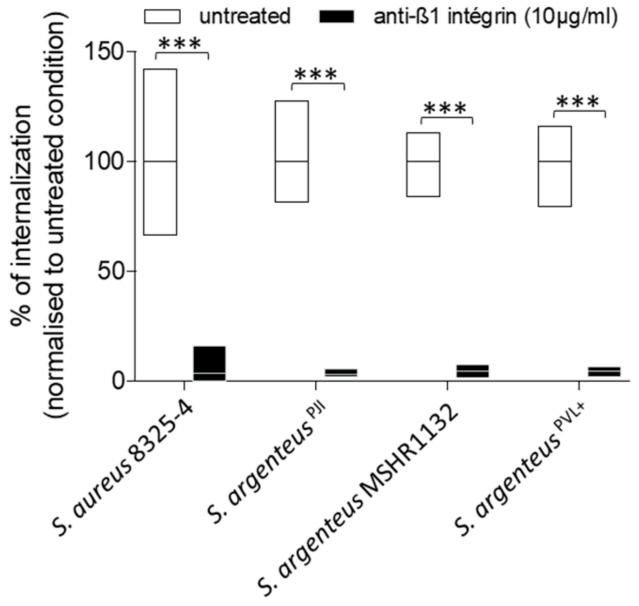
The internalization of *S. argenteus* strains into MG-63 cells requires β1 integrin. A pre-treatment of MG63 cells with a blocking anti-β1 integrin antibody inhibits more than 90% of *S. aureus* and *S. argenteus* strains internalization. Mann–Whitney test was performed for statistical analysis. *** means *p* < 0.001.

In osteoblastic cells, *S. aureus* is known to mainly use the β1-integrin as a receptor to trigger its internalization. In our study, treatment with blocking anti-β1 integrin antibodies resulted in a reduction of *S. argenteus* internalization by more than 95%, with a mean of 3.1% of internalization capacity left after treatment (Figure 3), which was comparable to *S. aureus* 8325-4 (Figure 3, 3.4% of internalization capacity left), suggesting that *S. argenteus* internalization pathway uses the same receptor or trigger.

Two other *S. argenteus* isolates tested showed similar results after anti-β1 integrin treatment with a mean of 4.7% of internalization capacity left after treatment (Figure 3).

Overall, these results demonstrate that all tested *S. argenteus* strains were able to invade osteoblasts using a β1 integrin-dependent mechanism.

### 2.3. Intracellular Survival

**Figure 4:**
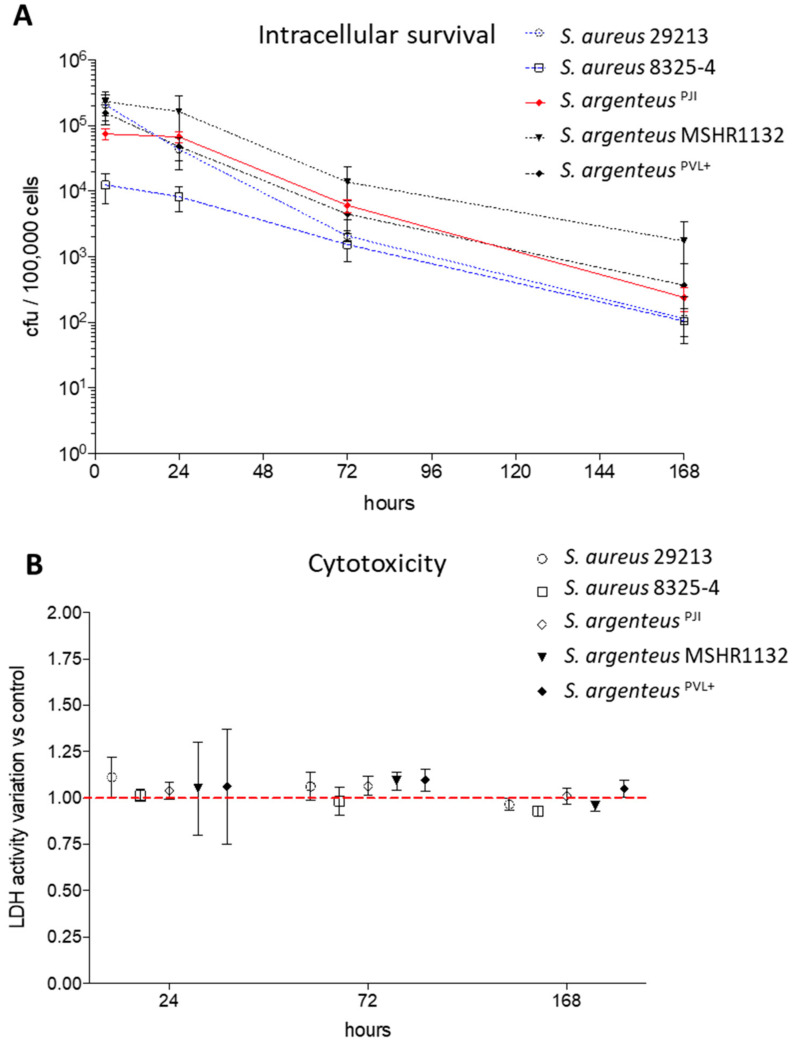
Intracellular survival and cytotoxicity induced by the *S. argenteus*^PJI^. (**A**) The intracellular inoculum of *S. aureus* (29213, 8325-4) and *S. argenteus* strains (*S. argenteus*^PJI^, *S. argenteus* MSHR1132, *S. argenteus^PVL+^*) was followed over seven days, expressed in CFU/100,000 cells, and showed a better persistence for the *S. argenteus* strains. (**B**) The cytotoxicity induced by the strains has been assessed by following the LDH release in the extracellular medium. The data are presented as variation when compared to the relative non-infected control for each tested time of infection No cytotoxicity is observed for any strain.

Interestingly, 24 h after internalization, the intracellular bacterial charge of the clinical *S. argenteus* isolate stayed similar to the one observed at 3 h post-infection (pi) (Figure 4A, 6.75 vs. 7.47 × 10^4^ CFU at 24 and 3 h pi, respectively).

After three days pi, the intracellular inoculum decreased by one log, reaching 6.07 × 10^3^ CFU. This bacterial load tended to be higher than those of the two *S. aureus* strains at the same timepoint (Figure 4A, 2.08 and 1.53 × 10^3^ CFU/100000 cells for 29213 and 8325-4 respectively). Focusing on *S. argenteus* strains, *S. argenteus*^PJI^ behaved similarly to *S. argenteus* MSHR1132, although the latter displayed higher intracellular inoculum after 24 and 72 h (Figure 4A, 1.64 × 10^5^ and 1.38 × 10^4^ CFU for MSHR1132 at 24 and 72 h). The *S. argenteus*^PVL+^, on the other hand, was not able to maintain its intracellular inoculum between 3 and 24 h pi (1.58 × 10^5^ and 4.36 × 10^4^ CFU at 3 and 24 h pi).

After seven days, a decrease of approximately 1-Log was observed when compared to intracellular inoculums at 72 h pi for all the strains (Figure 4A). However, intracellular bacteria were still detectable in all the conditions.

Altogether, these results point out that the *S. argenteus* isolates can survive intracellularly, as previously observed for *S. aureus*. However, the intensity of intracellular survival varies between the different *S. argenteus* strains. In our in vitro model, this could result either from a better intracellular life adaptation or from a decreased cytotoxic power that would limit the release of bacteria in the lethal extracellular environment that contains gentamicin.

### 2.4. Cell Death Induction by Intracellular Staphylococci

We evaluated the cytotoxic effect induced by intracellular bacteria by measuring the release of LDH at 24 h, 72 h and seven days after infection. Overall, we observed that the strains used in this study displayed a poor cytotoxic effect on non-professional phagocytic cells such as MG63, whether they are *S. aureus* or *S. argenteus* strains, expressing or not the PVL. After 24 h of infection, the cell death induced was still similar to what was observed for the invasive strain 29213 (Figure 4B, 104 ± 4% vs. 111 ± 11%, 101 ± 3%, 105 ± 25% and 106 ± 31% for *S. argenteus*^PJI^ vs. *S. aureus* 29213, *S. aureus* 8325-4, *S. argenteus* MSHR1132 and *S. argenteus^PVL+^,* expressed relatively to non-infected cells). The results were all in the same range at 72 h and seven days after infection (Figure 4B).

These results suggest that the better intracellular survival of *S. argenteus*^PJI^ is likely due to a better adaptation to the intracellular compartment.

### 2.5. Induction of a Cytokinic Response

We investigated the release of interleukin 6 (IL-6), a key cytokine in inflammation regulation, and granulocyte macrophage colony stimulating factor (GM-CSF), a stimulating factor for neutrophils and macrophages, by infected osteoblasts, once the infection was established and the bacteria were persistent in the in vitro set up, i.e., 72 h after infection.

**Figure 5:**
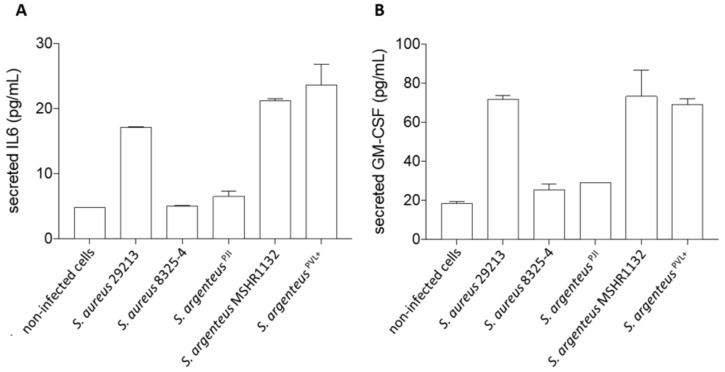
Cellular inflammation induced by the *S. argenteus*^PJI^ strain. The secretion of interleukin-6 (**A**) and GM-CSF (**B**) by MG63 cells was measured using ELISA assay. *S. argenteus*^PJI^ displays a low level of induced inflammation compared to the other invasive strains (*S. aureus* 29213, *S. argenteus* MSHR113*2* and *S. argenteus*^PVL+^).

First, we observed that *S. argenteus* MSHR1132 and *S. argenteus*^PVL+^ were among the strongest inducers of inflammation, with levels of secreted IL-6 similar or higher than the ones induced by the invasive *S. aureus* 29213 (Figure 5A, 21.3 and 23.6 vs. 17.2 pg/mL for *S. argenteus* MSHR1132 and *S. argenteus^PVL+^* vs. 29213).

Conversely, *S. argenteus*^PJI^ appeared as a weaker inducer of inflammation with a concentration of secreted IL-6 similar to the poorly invasive *S. aureus* 8325-4 (Figure 5A, 6.5 vs. 5 vs. 4.8 pg/mL for *S. argenteus*^PJI^ vs. *S. aureus* 8325-4 vs. non-infected cells).

In line with this weak induction of inflammation, *S. argenteus*^PJI^ induced a release of GM-CSF similar to *S. aureus* 8325-4 and closer to the levels observed for the non-infected cells (Figure 5B, 29.1 vs. 25.6 vs. 18.6 pg/mL for *S. argenteus* vs. 8325-4 vs. non-infected cells). GM-CSF release by osteoblasts infected by *S. aureus* 29213 or the two other *S. argenteus* was at least 2.5-fold higher (71.7 vs. 73.2 vs. 69 pg/mL for *S. aureus* 29213 vs. *S. argenteus* MSHR1132 vs. *S. argenteus^PVL+^*).

Together, these results suggest that *S. argenteus*^PJI^ induced a weak inflammatory response compared to the two other *S. argenteus* strains, which seems logical in a context of chronic and persistent PJI.

### 2.6. Assessment of the Ability to Form Biofilm

The other bacterial strategy used to shelter from the extracellular environment and antibiotics and persist is to form biofilm. First, we assessed the early biofilm formation capacity using the BioFilm Ring Test^®^ [26]. The results highlight no difference between strains, classifying the five tested strains as moderate early biofilm producers (Figure 5).

**Figure 6:**
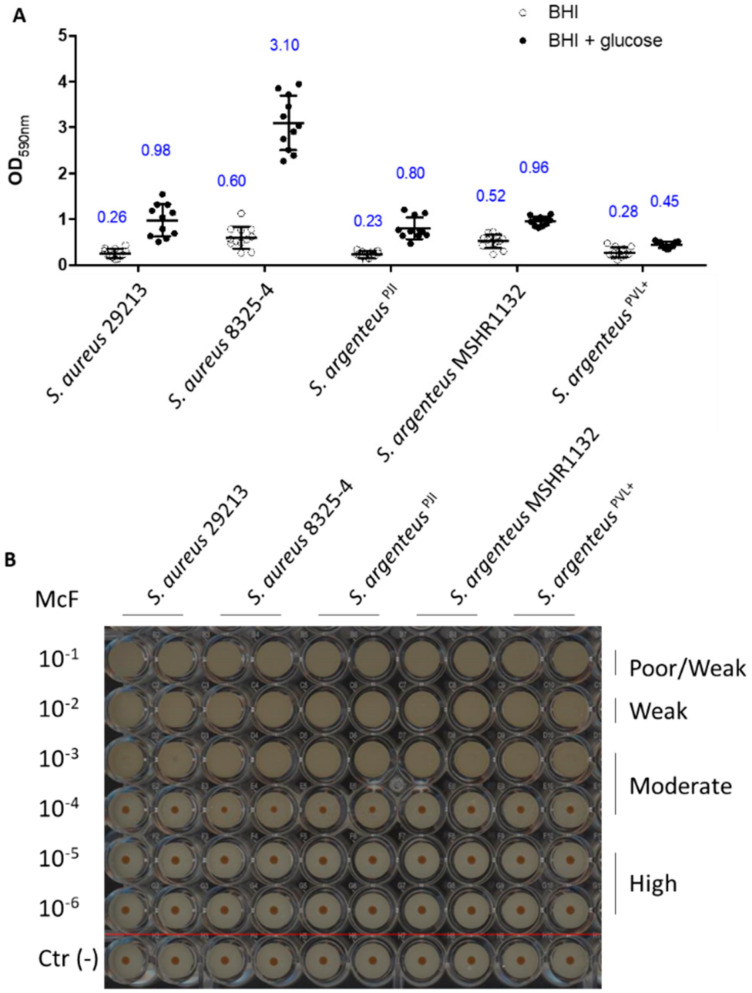
Biofilm formation capacities of the strains. (**A**) The biofilm formation after 24 h has been measured using OD measurement at 590 nm of the crystal violet staining (blue numbers are the OD_590_ values). (**B**) The early production of biofilm was assessed using the BioFilm Ring Test^®^ technology and classified based on the previously published procedure [26]. The image is representative of three independent experiments. Negative controls were performed for each condition (below the red line).

We then investigated the formation of mature biofilm (after 24 h) in absence or presence of glucose, using the crystal violet staining method. Indeed, addition of glucose in the culture medium promotes the formation of biofilm by *S. aureus,* especially MRSA strains [27]. In absence of glucose, *S. argenteus*^PJI^ was able to produce a mature biofilm in a way similar to the two *S. aureus* strains (Figure 6, OD_590_ = 0.23 vs. 0.6 vs. 0.26 for *S. argenteus* vs. *S. aureus* 8325-4 vs. *S. aureus* 29213).

The two other *S. argenteus* tested (MSHR1132 and *S. argenteus^PVL+^*) also showed an ability to form biofilm similar to the one of the *S. aureus* strains (Figure 6, OD_590_ = 0.52 and 0.28 for MSHR1132 and *S. argenteus*^PVL+^).

In the presence of glucose, *S. argenteus* isolates did not respond to a glucose stimulation as much as *S. aureus* strains, especially when compared to *S. aureus* 8325-4 (Figure 6A, OD_590_ = 0.8 vs. 0.98 and 3.1 for *S. argenteus* vs. *S. aureus* 29213 and *S. aureus* 8325-4; OD_590_ = 0.96 and 0.45 for MSHR1132 and *S. argenteus*^PVL+^).

Altogether, these results confirm another asset to the persistence ability of *S. argenteus*^PJI^, with a capacity to organize as a biofilm and protect itself from extracellular antimicrobials and the host immune system.

### 2.7. Genomic Analysis of Staphylococcus argenteus^PJI^

As stated above, the genome of *S. argenteus*^PJI^ was sequenced during a distinct research project in the lab and we took advantage of this to seek for clues on its abilities to persist inside cells and to form biofilm. Anticipated from its original description as being a MSSA, we did not find a SCC*mec* cassette in *S. argenteus*^PJI^ genome. However, we found a cassette in the two other *S. argenteus* strains, even if *S. argenteus*^PVL+^ is a methicillin-susceptible strain. In *S. argenteus*^PJI^, we found genes associated to antibiotic resistances for β-lactams, erythromycin and fosfomycin. As expected from the β1 integrin dependence of its internalization process, both *fnb* genes were present in the genome of *S. argenteus*^PJI^. Regarding virulence, we did not find the LukS-PV and LukF-PV subunits of the PVL toxin, nor any PSMα homologs in *S. argenteus*^PJI^. However, the PVL subunits were retrieved in the *S. aureus^PVL+^* strain. The presence of the *ica* operon (*ica*A, C and D), *gba*A and -B, *bbp* and the *psmβ* genes in all the *S. argenteus* was consistent with their ability to form biofilm.

**Figure 7:**
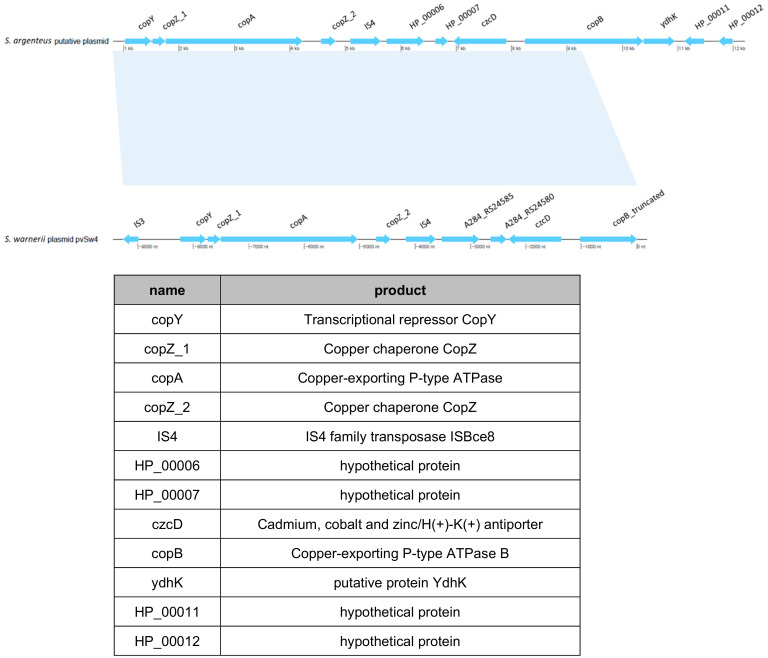
Identification of a putative plasmid in the *S. argenteus*^PJI^. A putative plasmid (top) was identified during the genome analysis, similar to the plasmid pvSw4 (bottom, NCBI access number: NC_020266.1) found in *S. warneri* [28]). The latter covers 73% and shares 99.48% of similarity with the one we identified in *S. argenteus* PJI. These plasmids encode for proteins mainly involved in copper or heavy metals transport (table).

One intriguing element was found in *S. argenteus*^PJI^ genome; it was located in the region where the SCC*mec* cassette is present in the two other *S. argenteus*. In this region, *S. argenteus* MSHR1132 and *S. argenteus*^PVL+^ possess a CRISPR element which is absent in *S. argenteus*^PJI^. Instead in *S. argenteus*^PJI^, we identified a functional region identified by BLAST as a copper-translocating P-type ATPase. This strain also harbored what seemed to be a putative plasmid bearing an operon that encodes proteins involved in copper and heavy metals transport or detoxification (Figure 7).

A similar plasmid (pvSw4, NCBI access number: NC_020266.1) was identified in *S. warneri* [28], which covers 73% and shares 99.48% of similarity with this one.

Finally, we also identified that the phage φSa3, inserted in the genome of the two other *S. argenteus* in the β-hemolysin region, is not present in *S. argenteus*^PJI^, which results in an intact β-hemolysin gene.

## 3. Discussion

We extensively describe here the first case of an insidious and chronic PJI caused by *S. argenteus* which happened after three episodes of MSSA infections and could finally be cured by a two-stage exchange strategy with prolonged antimicrobial therapy. Although all the staphylococcal strains identified during the first three episodes of infection were not available, we supposed that they might have been the same *S. argenteus* initially misidentified as *S. aureus*. This statement is reinforced by investigations of bacterial factors associated with BJI chronicity, including bone cell invasion and biofilm formation, revealing that this clinical isolate was well-equipped to be responsible for chronic infections.

The *S. argenteus*^PJI^ strain was able to invade cells in an efficient way, even if not as efficiently as the two other *S. argenteus* isolates or the invasive *S. aureus* 29213. Interestingly, although its intracellular inoculum was high after three days of infection, it induced only a low-grade inflammation. This ability to keep a low inflammatory profile seemed specific to this strain, as the two other *S. argenteus* strains induced a high inflammation. *S. argenteus*^PJI^ was also able to form biofilm in vitro and was classified as a moderate producer according to the procedure described in [26]. Altogether, these results suggest that *S. argenteus*^PJI^ harbors phenotypic characteristics associated with chronic BJI, combining non-phagocytic bone cell invasion and biofilm formation, with a very limited induction of the cellular inflammatory response. Moreover, the mechanism of bone cell invasion by *S. argenteus* likely relies on similar mechanisms that *S. aureus*, involving staphylococcal FnBP-like proteins and α5β1 cellular integrin, as suggested using blocking anti-β1 integrin antibodies.

Finally, the genome analysis revealed important information. First, we identified that this strain is equipped with proteins allowing to deal with copper or heavy metals detoxification or intake: a copper-translocating P-type ATPase in the genome as well as a putative plasmid encoding for ATPase copper exporter together with the related chaperones (copA, copB, copY and copZ) and proteins involved in the regulation of heavy metals resistance such as czcD [29]. This extra set of proteins required for copper tolerance could likely represent an effective strategy against the copper killing mechanisms [30,31,32] used by the host cells to eradicate the bacteria or, at the opposite, could help survive the copper limitation the host could use as another defensive mechanism [33]. Notably, it could play a role similar to the *copB* gene, *mco* operon and its repressor *csoR* identified on a mobile genetic element in methicillin-resistant *S. aureus* that increase survival in human blood and resistance to killing by host immune cells [34]. We also identified the presence of the β-hemolysin gene that has been involved in the suppression of the IL-8 production and in the inhibition of the neutrophil migration [35] adding to its low inflammatory potential.

In summary, both genetic features could participate to its good ability to survive in the host by resisting the host defenses and limiting the inflammation which would result in the chronicity of the infection.

Altogether, our study suggests that *S. argenteus* can be implicated in chronic PJI, as harboring phenotypic characteristics associated with immune response and antimicrobial escapement observed in persisting BJI. Based on our findings, we could make the hypothesis that an enhanced resistance to copper together with a propensity to keep a low profile in terms of inflammation induction could have bought this *S. argenteus*^PJI^ enough time inside the patient to invade cells or form biofilm. Once embedded in a biofilm or inside the intracellular compartment, the bacteria would have been better protected from the extracellular antibiotics and less easily accessible to intracellular antibiotics. This would have resulted in its persistence without clinical signs for the first six months of the episode and might explain the chronicity and the length of the anti-microbials treatments needed, as well as the requirement for prothesis ablation and reimplantation to get rid of this infection. Obviously, more work will be needed to test this hypothesis and identify the factors involved notably in the weak cellular response induced by this invasive *S. argenteus* PJI strain.

## 4. Materials and Methods

### 4.1. Cell Line and Bacteria Strains

The osteoblastic cell line MG63 (LGC standards, Molsheim, France) was used to explore adhesion, internalization, persistence of staphylococcal strains and the cellular response and cytotoxicity induced by the infection. In addition to *S. argenteus*^PJI^, the strain of interest isolated during the relapse of the PJI, we used 2 other strains of *S. argenteus*, including the methicillin-resistant strain type MSHR1132 [1,36] and a clinical PVL-positive methicillin-susceptible strain (*S. argenteus*^PVL*+*^) [20]. The *S. aureus* type strain ATCC 29213 and NCTC 8325-4, routinely used in our lab, were used as references.

### 4.2. Cell Culture Conditions

Cells were cultured in 75 cm^2^ flasks (T75, BD Falcon, Le Pont de Claix, France at 36 °C under 5% CO_2_, in a complete culture medium composed of DMEM (Dulbecco’s Modified Eagle Medium, 31885, containing D-glucose, L-glutamine, pyruvate) supplemented with 10% fetal calf serum (FCS, 10270106), penicillin (100 μg/mL) and streptomycin (100 μg/mL), all from Gibco (Paisley, UK). Cells were passaged once a week and used until passage 25 at most.

### 4.3. Infection and Gentamicin Protection Assay

MG63 cells were seeded at 100,000 cells per well in a 24-well plate and incubated for 24 h, before being infected at a MOI of 100:1 with the different strains. After 2 h of contact, cells were washed twice and incubated for 1 h with a high concentration of gentamicin (200 µg/mL) in order to eliminate adherent and non-adherent extracellular bacteria. For the persistence assessment, the media was replaced with the same fresh media supplemented with gentamicin (40 µg/mL) (to prevent possible reinfection of new cells after lysis of infected cells liberating intracellular bacteria), and changed every 48 h except for the cytokine secretion measurement (see below). Cells were lysed by osmotic shock in water after 2 h, 3 h, 24 h, 72 h and 7 days, and lysates were plated on TSA agar plates using the Easy Spiral^®^ automaton (Interscience, Saint-Nom-la-Bretèche, France). CFU were counted after 18 h of incubation at 36 °C. Bacterial adhesion was calculated by subtracting the CFU counted at 3 h to those counted after 2 h.

### 4.4. Cell Death Assessment

The cell death was evaluated by the quantification of the lactate dehydrogenase (LDH) released in the extracellular medium using the Cytotoxicity Detection kit (Roche, Basel, Switzerland, 11644793001) following the manufacturer recommendations.

### 4.5. Cytokines Secretion by Infected Osteoblasts

GM-CSF and Il-6 secretions were quantified in the cell culture supernatant after 72 h of infection without media change, using the human GM-CSF and IL-6 DuoSet ELISA kit from R&D Systems (refs. DY215 and DY206) and according to the manufacturer’s recommendations.

### 4.6. Biofilm Formation

Early biofilm formation capacity was tested using the BioFilm Ring Test^®^ technology (BioFilm Control, Saint Beauzire, France), a magnetic bead immobilization assay which allows the classification of strains according to the previously published procedure [26].

Mature biofilm production was quantified using crystal violet staining associated with the steam-based washing method (BiofilmCare) as described previously [37].

### 4.7. Whole Genome Sequencing

Genomic DNA was extracted from each isolate using a QIAcube extraction kit (Qiagen, Hilden, Germany) and sequencing libraries were prepared from 1 ng of DNA using the Nextera XT DNA preparation kit (Illumina). Whole-genome sequencing was done with an Illumina MiSeq (Illumina, San Diego, CA, USA) to generate 300-bp paired-end reads that were de novo assembled using Unicycler v 0.4.5 pipeline [38] with default parameters (including read’s correction and trimming steps before the assembly, scaffolding, removing overlap and bridging steps). A quality assessment of the assemblies was performed with QUAST v 4.6.3 [39].

The genome of the chronic *S. argenteus* clinical isolate was compared with those of the *S. argenteus* type strain MSHR1132 [1,36] (GenBank acc. num. FR821777,2), a previously described PVL+ strain [20] and the *S. aureus* strain NCTC8325 (GenBank acc. num. NC_0077951).

The presence or absence of 38 *S. aureus* genes known to be involved in internalization, biofilm production, persistence and virulence was investigated using ABRicate v 0.8.1 [40] with default parameters (Table 2).

The resistome of all *S. argenteus* genomes was assessed with SRST2 using the ResFinder [41] database. The characterization of SCC*mec* and CRISPR elements were performed, respectively, with SCCmecFinder [42] and CRISPRcasFinder [43]. The presence of the small phenol soluble modulins alpha 1–4 (PSMα1–4) was assessed by tblastn using the protein sequences of MSHR1132 PSMα as query. The genome annotation was made with Prokka v 1.13.3 [44], and Mauve progressive [45] alignments were used to check the location of the annotated leukocidins. The differential presence of genes between S. *argenteus* and *S. aureus* and between the chronic *S. argenteus* and the other *S. argenteus* was assessed with Roary v3.11.2 [46]. The presence of phage was tested using Phaster [47].

### 4.8. Study Protocol and Statistical Analysis

For phenotype experiments, three independent experiments were performed for each read-out in technical triplicate (3 wells for each conditions) except for cytokine secretion measurements that were only performed on supernatants from 2 independent experiments. For internalization and persistence assays, results are presented as the number of intracellular staphylococci for 100,000 osteoblastic cells. Due to the observational nature of this study, we chose to not perform statistical analysis, except for the results in Figure 3.

## Figures and Tables

**Table 1:** Antibiogram of the *S. argenteus^PJI^* strain. R is for resistant and S is for sensitive according to the CASFM guidelines.

Antibiotic	Susceptibility	Antibiotic	Susceptibility	Antibiotic	Susceptibility
Peni-G	R	Erythromycin	R	Vancomycin	S
Peni-M	S	Lincomycin	R	Teicoplanin	S
Gentamicin	S	Synergistins	S	Fosfomycin	S
Tobramycin	S	Rifampicin	S	Fusidic acid	S
Kanamycin	S	Nitrofurantoin	S	Linezolid	S
Tetracyclin	S	Cotrimoxazol	S		
Minocyclin	S	Ofloxacin	S		

**Table 2:** Presence (+) or absence (−) of genes involved in *S. aureus* virulence, in the *S. argenteus* strains used.

	*S. argenteus* ^PJI^	*S. argenteus* MSHR1132	*S. argenteus* ^PVL+^
**PSM genes**			
PSM_alpha1	+	+	+
PSM_alpha2	+	+	+
PSM_alpha3	+	+	+
PSM_alpha4	+	+	-
PSM_beta1	+	+	+
**Biofilm formation**			
ebpS	+	+	+
icaA	+	+	+
icaB	+	+	+
icaC	+	+	+
fib	+	+	+
eno	+	+	+
bbp	+	+	+
clfA	+	+	-
clfB	+	+	-
**Internalisation**			
fnbpA	+	+	+
fnbpB	+	+	+
atl	+	+	+
clfA	+	+	+
sdrD	+	+	-
tet38	+	+	+
sraP	+	+	-
eap	+	+	+
gapC	+	+	+
**Persistence**			
sdhA	+	+	+
sdhB	+	+	+
ureG	+	+	+
mnhG	+	+	+
fbaA	+	+	+
ctaB	+	+	+
mazF	+	+	+
glpX	+	+	+
clpX	+	+	+
parE	+	+	+
**Leukocidins**			
hglA	+	+	+
hglB	+	+	+
hglC	+	+	+
**Virulence**			
sigB	+	+	+
agrA	+	+	+

## Abbreviations

BJI	Bone and Joint Infection
CFU	Colony Forming Unit
FnBP	Fibronectin Binding Protein
GM-CSF	Granulocyte Macrophage Colony Stimulating Factor
LDH	Lactate Deshydrogenase
MRSA	Meticillin Resistant *Staphylococcus aureus*
MSCRAMM	microbial surface components recognizing adhesive matrix molecule
MSSA	Meticillin Susceptible *Staphylococcus aureus*
NPPC	Non Professional Phagocytic Cell
PJI	Prosthetic Joint Infection
PSM	Phenol Soluble Modulin
PVL	Panton Valentine Leukocidin
SCV	Small Colony Variant
